# Seasonal variations in the diagnosis of childhood cancer

**DOI:** 10.1054/bjoc.2000.1343

**Published:** 2000-08-16

**Authors:** D Machin, F Gao

**Affiliations:** Division of Clinical Trials & Epidemiological Sciences, National Cancer wCentre, 11 Hospital Drive, 169610, Singapore; School of Health and Related Research, University of Sheffield, Northern General Hospital, Herries Road, Sheffield, S5 7AU, UK


*British Journal of Cancer* (2000) **83**(5), 699–700
© 2000 Cancer Research Campaign
doi: 10.1054/ bjoc.2000.1343, available online at http://www.idealibrary.com on IDEAL®

Letter to the Editor

### Seasonal variations in the diagnosis of childhood cancer

**Sir**

Ross and colleagues (Ross et al, 1999) describe in their Table 1 the seasonal variation in the diagnosis of 12 childhood cancers in the USA but quote the peaks only in terms of seasons (see comment by Machin and Chong, 1999). However, the techniques reviewed by Machin and Chong (1998) can provide an estimate of the date of peak presentation, an appropriate confidence interval (CI) and (somewhat related) a measure of the strength of that peak (*R*); with minimum value 0 and maximum 1.

We have re-examined the data along these lines and the calculations are summarized in our Table 1 (because the data are only available grouped by month there is spurious precision in quoting the actual day). The disease-specific results are now presented in the calendar order of the respective peak days observed. In all the childhood cancers of Table 1 *R* is small (ranging from 0.016–0.140) and CIs rather wide, giving little support to important epidemiological effects.

In addition, the peak date allows easier comparisons to be made across studies. As an illustration, the peak in children with NHL in the USA may be compared with the peak reported by Westerbeek et al (1998, Table 3) for the UK. The peak for USA patients is indicated as 23 June (more sensibly the June/July period) and this contrasts rather markedly with the estimate of 19 February (95% CI 5 September to 9 August) for those reported by Westerbeek and colleagues (see our Table 1). However, the wide CIs and small *R* both suggest little support for any distinct peak. Thus the observed 4-month difference in peak presentation may merely be a chance difference. However, these two dates may be compared using the regression technique for directional data described by Fisher (1993). This estimates the interval between these two observed peaks, which can then be tested under the null hypothesis of a common date. Not surprisingly, this lead to a non-statistically significant difference ($P$ = 0.80, $R$ = 0.017) for this comparison.

We would question whether it is truly meaningful to summarize the 'overall' pattern by summing the monthly counts of the $k$ = 12 cancers as Ross et al (1999) have done. The more common tumours will dominate such totals and this process also assumes that there is indeed a common peak for these cancers. We suggest that it may be better to summarize this information by calculating the 'overall' peak from the 12 individual disease specific peak dates of Table 1, Column 3. This estimates a relatively strong peak, $R$ = 0.273, at 14 June but an associated wide 95% CI.

In principle, the Fisher methodology can be extended to provide a better summary of these 'overall' data with CIs taking into account the sizes of the individual tumour groups. It can also be used to investigate the influence of latitude alluded to by Ross et al (1999).

Ross and colleagues also list 19 other studies that have explored childhood cancer and seasonality and, from their Table 2, one can see that a wide range of statistical approaches have been used to summarize these. However, the synthesis of these studies is made in purely descriptive terms and from which the authors conclude, for example, '… provides some modest support for a summer excess in the diagnosis of childhood ALL'. In other situations, particularly in the context of randomized trials, overviews of studies using formal meta-analytic techniques have provided a useful synthesis of the data. In principle, the regression model could synthesize relevant seasonality studies through a formal meta-analysis. Unfortunately, the calculations are complex and computer packages are not available for their implementation, although we are currently developing procedures for some of these purposes.

**Table 1** Estimated peak date of presentation of childhood cancers, corresponding 95% confidence intervals

| Cancer type | Patients (*n*) | Peak date | 95% CI | *R* | *P* |
|---|---|---|---|---|---|
| Ross et al (1999) | | | | | |
| CNS | 3855 | 18 Jan | 13 Oct to 06 Mar | 0.026 | 0.073 |
| NB | 1495 | 26 Feb | 08 Nov to 01 Jul | 0.024 | 0.42 |
| RD | 861 | 18 Apr | 07 Mar to 19 Jun | 0.065 | 0.026 |
| WT | 1245 | 11 Jun | 19 Apr to 09 Aug | 0.045 | 0.077 |
| ES | 437 | 17 Jun | 25 Jan to 06 Nov | 0.030 | 0.67 |
| NHL | 1325 | 23 Jun | 25 Jan to 23 Nov | 0.016 | 0.74 |
| AML | 1153 | 03 Jul | 26 Mar to 24 Oct | 0.031 | 0.33 |
| ALL | 5532 | 09 Jul | 17 May to 14 Aug | 0.029 | 0.009 |
| HB | 228 | 09 Jul | 29 May to 19 Aug | 0.140 | 0.012 |
| OS | 776 | 13 Sep | 16 Jun to 09 Jan | 0.037 | 0.34 |
| HD | 1142* | 27 Nov | 20 Jun to 28 Apr | 0.015 | 0.78* |
| Rb | 402 | 27 Dec | 22 Jul to 11 Jun | 0.026 | 0.76 |
| Overall | $n$ = 20 949 | 08 Jun | 15 Apr to 14 Aug | 0.010 | 0.13 |
| | $k$ = 12 | 14 Jun | 19 Jan to 19 Oct | 0.273 | 0.41 |
| Westerbeek et al (1998) | | | | | |
| NHL | 189 | 19 Feb | 05 Sep to 09 Aug | 0.020 | 0.93 |

* Given as 1135 and 0.62 respectively by Ross et al (1999)

In the meantime, we suggest that all studies reporting on seasonality should utilize individual patient data, provide estimates of the day of the peak date with a CI and report on magnitude.

*D Machin[1,2] and F Gao[1]*
*[1]Division of Clinical Trials & Epidemiological Sciences, National Cancer Centre, 11 Hospital Drive, Singapore 169610; [2]School of Health and Related Research, University of Sheffield, Northern General Hospital, Herries Road, Sheffield S5 7AU, UK*

**REFERENCES**

Fisher NI (1993) *Statistics of Circular Data*. Cambridge University Press: Cambridge
Machin D and Chong SF (1998) On the detection of the seasonal onset of disease. *J Epid Biostats* **3**: 385–394
Machin D and Chong SF (1999) Seasonal variations in the presentation and growth of thyroid cancer. *Br J Cancer* **79**: 1626–1627
Ross JA, Severson RK, Swensen AR, Pollock BH, Gurney JG and Robison LL (1999) Seasonal variations in the diagnosis of childhood cancer in the United States. *Br J Cancer* **81**: 549–553
Westerbeek RMC, Blair V, Eden OB, Kelsey AM, Stevens RF, Will AM, Taylor GM and Birch JM (1998) Seasonal variations in the onset of childhood leukaemia and lymphoma. *Br J Cancer* **78**: 119–124

doi: 10.1054/ bjoc.2000.1377, available online at http://www.idealibrary.com on IDEAL®

### Seasonal variations in the diagnosis of childhood cancer – reply

We thank Machin and Gao for their interest in our paper exploring seasonal variations in the diagnosis of childhood cancer. As they note, different statistical tests have been used to evaluate seasonal variations, and although these tests are statistically valid, they might not make full use of the data. We appreciate Machin and Gao's application of the Fisher method to our data. The Fisher method does provide a bit more precision than Roger's test (including an estimate of the strength of the peak). The conclusions of our paper, however, remain. Moreover, it is unclear how clinically useful it is to identify a peak that occurs on a specific day rather than within a season. Finally, we agree with Machin and Gao that a formal meta-analysis of all papers describing seasonality in the diagnosis of childhood cancer may be helpful; we look forward to when this analysis is conducted.

*JA Ross and RK Severson*
*Division of Epidemiology/Clinical Research,*
*Department of Paediatrics,*
*University of Minnesota*
*Medical School*
*Minneapolis, USA*

© 2000 Cancer Research Campaign

